# Video observation in HIT development: lessons learned on benefits and challenges

**DOI:** 10.1186/1472-6947-12-91

**Published:** 2012-08-22

**Authors:** Anna Marie Høstgaard, Pernille Bertelsen

**Affiliations:** 1 Department of Development and Planning, Virtual Centre of Health Informatics, Aalborg University, Fibigerstræde 13, 9220, Aalborg Ø, Denmark

**Keywords:** Health information technology, Video observation, Visual ethnography, Visual methodology, Sociomateriality, Clinical work practice studies, Work practice studies

## Abstract

**Background:**

Experience shows that the precondition for the development of successful health information technologies is a thorough insight into clinical work practice. In contemporary clinical work practice, clinical work and health information technology are integrated, and part of the practice is tacit. When work practice becomes routine, it slips to the background of the conscious awareness and becomes difficult to recognize without the context to support recall. This means that it is difficult to capture with traditional ethnographic research methods or in usability laboratories or clinical set ups. Observation by the use of the video technique *within* healthcare settings has proven to be capable of providing a thorough insight into the complex clinical work practice and its context - including parts of the tacit practice. The objective of this paper is 1) to argue for the video observation technique to inform and improve health-information-technology development and 2) to share insights and lessons learned on benefits and challenges when using the video observation technique *within* healthcare settings.

**Methods:**

A multiple case study including nine case studies conducted by DaCHI researchers 2004–2011 using audio-visual, non-participant video observation for data collection *within* different healthcare settings.

**Results:**

In HIT development, video observation is beneficial for 1) informing and improving system design 2) studying changes in work practice 3) identifying new potentials and 4) documenting current work practices.

**Conclusions:**

The video observation technique used within healthcare settings is superior to other ethnographic research methods when it comes to disclosing the complexity in clinical work practice. The insights gained are far more realistic compared to traditional ethnographic studies or usability studies and studies in clinical set ups. Besides, the data generated through video recordings provide a solid basis for dialog between the health care professionals involved. The most important lessons learned are that a well considered methodology and clear formulated objectives are imperative, in order to stay focused during the data rich analysis phase. Additionally, the video observation technique is primarily recommended for studies of specific clinical work practices within delimited clinical settings. Overall, the video observation technique has proven to be capable of improving our understanding of the interwoven relation between clinical work practice and HIT and to inform us about user requirements and needs for HIT, which is a precondition for the development of more successful HIT systems in the future.

## Background

Health Information Technology (HIT) plays an essential role in the delivery of health care today. However, the history of HIT shows that HIT projects are not only beneficial, but often fail to meet their goals [1-3] despite, - as stated by Kaplan et al. - “*an accumulation of best practice research identifying success factors*” [4] p.291. Studies show that in HIT development many of the failures, besides budget and timeline overruns, are due to problems of an organizational nature, resulting in investments in health information technologies without gaining the expected benefits [3,5,6]. Studies also show, that one of the reasons is the system developer’s lack of understanding of the complexity of clinical work practice [7-9]. Because the clinical work practices are enacted and by nature fundamentally social, clinicians like other human beings have “*an extraordinary ability to ‘make do’ with the technology with which they are provided*” [10] p. 435. This indirectly hinders the expected clinical benefits to mature. Thus, a precondition for the development of successful health information technologies, that provide clinical benefits, is that HIT development and implementation is based on a thorough insight into clinical work practice. A socio technical research approach contributes to this.

Research within DaCHI (Danish Centre for Health Informatics^a^) at the Department of Development and Planning at Aalborg University, Denmark shows that in contemporary clinical work practice, clinical work and health information technology are closely integrated, and that they should, when studied, be viewed as mutually dependent and not as discrete entities [9,11-24]. This view is supported by Orlikowski and Suchmann, who have introduced the concept of “Sociomateriality”, which has the notion that there is an inherent inseparability between people and technology in organizational work [25,26]. Additionally, part of the clinical sociomaterial work practice is of a tacit nature, because when work practice becomes routine (know-how), it slips to the background of the conscious awareness and becomes difficult to recognize without having the context to support recall [10,13,27,28]. This stipulates specific demands when studied, because tacit knowledge is difficult to pass on to others (in words or in writing) with traditional ethnographic data collection techniques such as personal observation and interviews.

Realizing this dilemma, researchers in social sciences already more than three decades ago introduced video observation as a data collection technique capable of providing insight into sociomaterial work practice – often used in combination with other ethnographic data collection techniques [29]. Since then, the use of video observation has become still more widespread within disciplines of social anthropology (visual anthropology) and sociology to study people’s behavior and their social interactions in a number of different contexts [30-35].

In HIT development, the potentials of video observation, as a technique capable of capturing data that provide insight into part of the tacit interactions between health care professional and health information technology, is still far from being exploited. Today most studies of clinical work practice are still performed by traditional ethnographic data collection techniques or by usability studies in clinical set ups^b^ or in laboratories [36-39]. When video observation has been used within healthcare settings (at hospital wards) it has been to study specific clinical procedures (e.g. handover of surgical instruments in surgical theatres) [40,41], for studies of social processes between different clinical professional groups [42], or in order to study the impact of the video observation technique on the clinicians by asking them to comment on their work in front of the camera: ie. “video as a means of reflection and elicitation” [43] p.16.

Traditional ethnographic data collection techniques provide so to speak an insight into “what the clinicians *think* they do” and “what they *say* they do” during their daily clinical work. Recognizing that using these techniques give little access to information on “what clinicians *actually* do” when they perform their daily clinical work - often in cross disciplinary teams, researchers within DaCHI have during recent years focused on the development of new methods and techniques capable of giving an answer to this essential question.

Since 2004 we have studied clinical work practice, using video observation *within* the healthcare sector, ie. at hospital wards. Through these studies we have found that video observation of clinicians, performing their daily tasks, provide a thorough insight into the clinical sociomaterial work practice - including parts of the tacit work practice [12,16-19,22,24,44,45].

The importance of continuing research in this area is supported by recent studies in HIT systems success and failures. They stress a need for more research into the development of new research methods and techniques for achieving a better understanding of clinical work practice, and hence as a mean to develop successful health information technologies [4,5].

The objective of this paper is therefore, to argue for the use of the video observation technique to inform and improve health information technology development and when doing so, to share our experiences and learned lessons on benefits and challenges when using the video observation technique *within* healthcare settings, ie. at hospital wards.

## Methods

### Study paradigm

Depending on the study paradigm (e.g. hermeneutical, phenomenological, positivistic) [46] and the objective (e.g. evaluation, gaining insight into a specific context, practice or documentation) researchers take different ontological points of views on the study topic. Awareness of this fact when preparing the study methodology, as well as being explicit about it, when presenting the study results, need to be stressed, as it is a precondition for valid and reliable study results [34,47-49].

The views, knowledge and experiences presented in this paper are based on a hermeneutical point of view on the clinical sociomaterial work practice. In the specific context of the healthcare sector, this means that we try to understand and to interpret “what the clinicians are doing”, when they perform their daily clinical work. Thus, our research aims to provide a basis for understanding the processes that cause an intervention (HIT development and implementation) to make a difference in a specific context (the healthcare sector) [46]. One of the factors characterizing the hermeneutical study paradigm is that the relevant actors have to be identified, in order to interpret their opinions on the process and on the results. Our socio-technical approach to studies of the sociomaterial interactions between humans and technology in HIT development is fully in line with this [9,11,17,50].

However, the fact that the views presented in this paper are based on a hermeneutical study paradigm does *not* mean that the video observation method cannot provide benefits within other study paradigms. One of the cases included in this paper is an example of this, as the objective is both of an explanatory (positivistic) and an understanding / interpreting (hermeneutical) nature [12].

### Methodology

Below a short overview on the study methodology is first presented. Then, the different aspects are presented in more details:

Theoretical approach: Socio-Technical

Design: Multiple case

Study period: 2004–2009

Study object: The sociomaterial interactions between users and technology in clinical work practice

Data collection method: Ethnographic

Data collection technique: Observation

Data collection instrument: Video

Data analysis and trustworthiness: See below

### Theoretical approach

This study is based on a socio-technical approach to studies of the sociomaterial interactions between humans and technology in HIT development. According to this, the implementation of HIT will lead to a mutual and sustained interaction between and transformation of the involved users/organization by the technology, and of the technology by the involved users/organizations. Embedded into the approach is a user-oriented perspective, [7,51].

### Design and study period

The study is a multiple case study, as the experiences presented are based on the results of nine case studies conducted by DaCHI researchers 2004–2011 using audio-visual video observation techniques for data collection *within* different healthcare settings – ie. at different hospital wards. The nine cases are listed below.

### DaCHI research cases 2004–2011

1. Recycling of Administrative Patient Data to measure Health Professional Quality, 2011 [12]

2. Evaluation of IT support for the Common Acute Receiving Unit at Horsens

Regional Hospital, 2010 [18]

3. Evaluation of the ‘Clinical Process' Electronic Health Record (EHR) in the

Danish Northern Region, 2010 [44]

4. Usability of CPOE (Computer Physician Order Entry System) 2010 [19]

5. Studies of clinical work practice illustrated through three cases, 2007 [24]

6. Medical Secretaries work practice before the implementation of the EHR, 2005 [16]

7. User-influence on the organization of work in a hospital ward before and after the implementation of the EHR, 2004 [17]

8. Management of surgical programs, 2004 [22]

9. Evaluation of the GEPKA project, 2004 [45]

A common characteristic on these case studies is that the video-data were all audio-visual and non-participant.

In Table 1, the four most recent studies are presented for a more detailed presentation on the kind of studies that we have dealt with in DaCHI 2004–2011. Thus, Table 1 shows an overview of the objectives, design, context and video observation technique, as well as the complementary methods of the cases numbered 1–4 above.

**Table 1:** Overview of the methodology in the four most recent DaCHI studies

**Case nr.**	**Objectives**	**Design**	**Context**	**Video observation technique**	**Complementary data collection methods**
**1**[12]	Assessment of the laborsaving effect by recycling administrative data. Focus on data-entry	a) Quantitative before/after evaluation - time-study	8 hospital wards at 5 different hospitals	2 researchers, each of them observing the data-entry with and without recycling administrative data for app. 3 hours at each ward	Interviews with the ward management and the nurses responsible for entering data into the systems at each ward
	Assessment of time consumption before and after data-entry	b) Qualitative ethnographic evaluation study			
**2**[18]	Assessment of the clinical benefits of implementing IT-boards for better overview with focus on the clinicians points of view	Qualitative ethnographic evaluation study	1 hospital ward	2 researchers, each of them observing the work practice after the implementation of the IT-boards for 1 full day (app. 7 hours)	Interviews with ward management
**3**[44]	Assessment of the clinical benefits by implementing EHR in a region in Danmark – focus on the clinicians points of view	a) Qualitative and quantitative* before/after evaluation study	5 hospital wards at 4 different hospitals	3 researchers, each of them observing 1 full day (app. 8 hours) at each ward before implementation and 1 full day (app. 8 hours) at each ward after implementation	Personal observation, Interviews with ward management and 2 physicians and 2 nurses selected by management at each ward. Questionnaires, Insight into documents
	Continuous feed-back on the process to the project management	b) Process evaluation			
**4**[19]	Creation of a basic understanding of the medication process in a cardiology department	Qualitative survey study	1 hospital ward	2 researchers each of them observing for a full day (app. 7 hours)	Photo supported interviews (photos taken by respondents)

*The quantitative objective in case nr. 3 was pursued by questionnaires – and not by video observation.

### Study object

The study object is the sociomaterial interactions between the clinicians and the HIT in clinical work practice.

### Data collecting method, technique and instrument

The data collecting method, technique and instrument focused on in this study is ethnographic observation by video. All video observations have been audio-visual. This means that data on the sociomaterial interactions between the clinicians and the technology in clinical work practice have been collected in both picture and sound. Besides, all video observations have been non-participant, meaning that the researcher has had no/minimum interactions with the clinicians and the clinical work practice during data collection.

**Figure 1:**
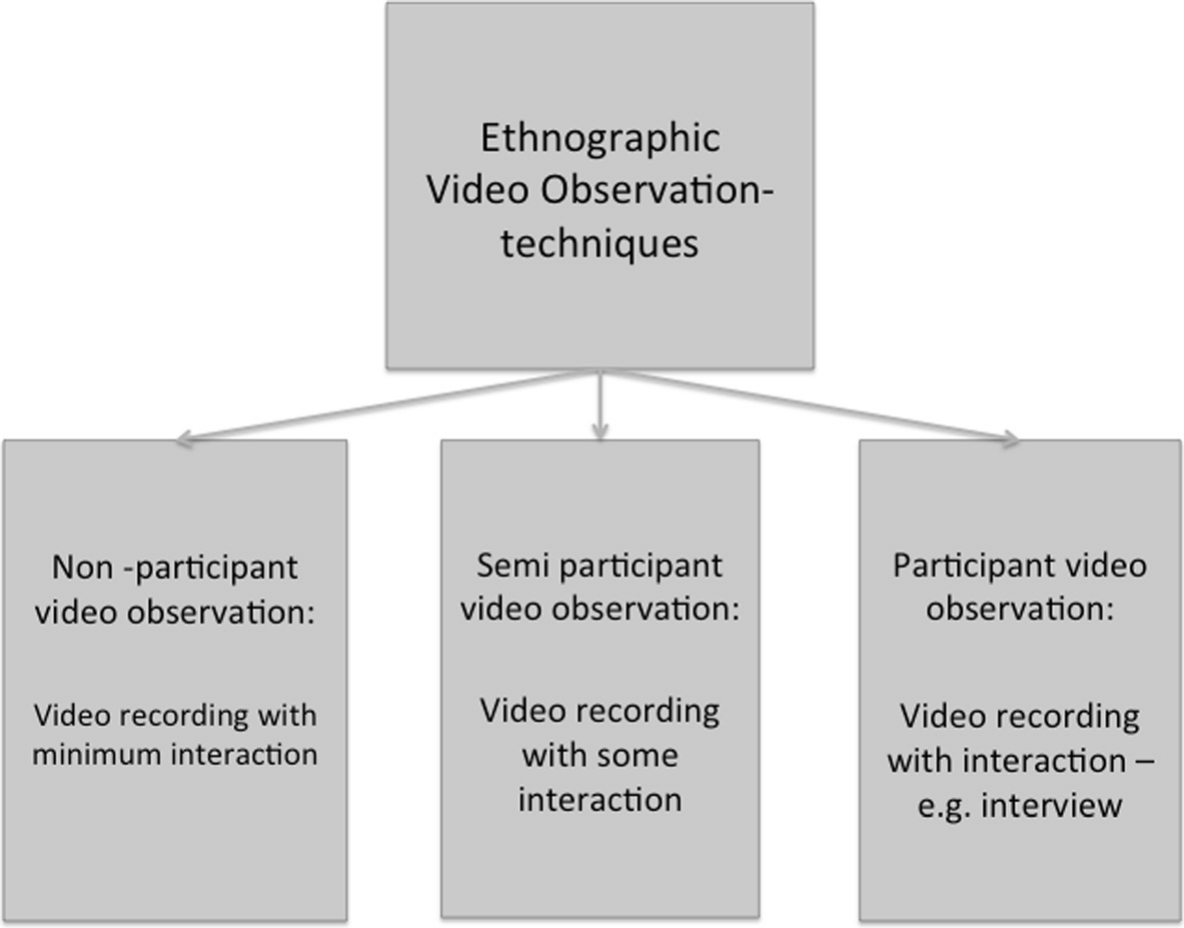
**A conceptual framework on ethnographic video observation techniques.** Figure 1 shows the conceptual framework on different ethnographic video observation techniques.

It is important to stress that we do not consider the video observation technique itself a research *method*. Video observation is one out of more observational data collection techniques within ethnographic data collection methods, ranging from non-participant video observation to participant video observation [52-54] (Figure 1). In contrast to data generated by personal observation, data generated by video observation are most often both visual and audial [47]. 

Other ethnographic methods as personal observation and interviews have also been used. However, they have served mainly to provide an insight into the hospital/ward logistics and organization (number of wards, employee, shifts etc.), hence as a preparation for the video observations.

### Data analysis and trustworthiness

First, the video sequences relevant according to the study objective have been identified. Next, these sequences have been transcribed and mapped. The interpretation process has been done with a focus on the objective. Trustworthiness has been sought by validating the interpretations by the clinicians participating in the recordings and by thoroughly describing all activities throughout the process (transparency).

### Ethical considerations

According to the Danish “Law on the Scientific Committee System and the treatment of biomedical research, chapter 3, paragraph 8, section 3”, a formal ethical approval from “The Danish National Committee on Research Ethics” is only required, if the study includes human biological materials. However, according to the law “Danish Act on Processing of Personal Data” chapter 4, paragraph 6, section1”, informed consent is required from all patients and staff-members, who are video-recorded. Whether this should be in oral or in writing is not specified in the law as stated above. Out of informal ethical considerations, we (researchers within DaCHI) try to avoid to video record patients – and especially patients faces. If a patient’s face by accident is video recorded, we delete the sequence.

For staff-members, we (researchers within DaCHI) have decided on obtaining informed consent in *oral* for video-recordings only to be studied by the researchers. For video-sequences to be shown to others at, e.g. scientific meetings, we obtain informed consent in *writing* from all staff participating in the video.

## Results

The results presented in this paper are based on the *total* experiences from 9 case studies. The individual results are not presented, as the objective of this paper is to argue for the relevance of the video observation technique to provide insight into sociomateriality and by doing so inform and improve health information technology development. Further, the aim is to share our experience on the most important lessons learned on benefits and challenges when using the video observation technique *within* healthcare settings – and not to present the results of the individual cases, which have quite different objectives (e.g. assessment of clinical benefits and labor-savings when implementing HIT).

During our studies, we have developed a generic guideline on how to conduct a video observation study:

1. contact to the management at hospital level (explain objective and methods)

2. contact to the management at ward level (explain objective and methods)

3. preliminary visits to wards (information to clinicians and other relevant actors, studies of logistic and organizational issues by personal observations and interviews)

4. video observation

5. data-analysis and interpretation

6. data validation with clinicians and other relevant actors

7. data presentation

Besides, we have gained thorough experiences on where, when and how to use video observation to inform and to improve HIT development. Based on our experiences, video observation provides benefits in studies with the following objectives:

1. to *inform and improve* the design of new health information technologies through studies of specific clinical work practices in delimited clinical settings

2. to *study changes* of specific clinical work practices before and after the implementation of new health information technologies in delimited clinical settings

3. to *identify potentials* for new ways to organize clinical work practice - including potential labor savings - when implementing new health information technologies through studies of specific clinical work practices in delimited clinical settings

4. to *document* current clinical work practice for future research purposes (e.g. before and after new HIS is implemented) through studies of specific present clinical work practices in delimited clinical settings

A number of benefits are common to the study objectives listed above - as well as a number of lessons learned on how to manoeuver as a researcher collecting data by use of video observation *within* healthcare settings.

### Common benefits

During our studies, we have found that video observation is most beneficial for studies of specific work practice within delimited clinical settings. Trying to study a wide range of clinical work practice at a number of different clinical settings at once is extremely time consuming and should only be done if based on thorough considerations on why and how to conduct the study.

Used within the healthcare sector, video observation permits us to explore *context dependent* clinical sociomaterial work practice, often involving staff with different professions in contrast to in usability laboratories or in clinical set-ups. In the latter unforeseen disruptions and communication challenges can be hard to imitate no matter how realistic the clinical setting has been set up [36-39].

In a video observation study on the impact of the Electronic Health Record (EHR) on sharing information’s between nurses at morning meetings, the nurses sought information’s in two different EHR systems, while at the same time making handwritten notes at other schemes and papers. The interactions between the nurses and the different artifacts went on extremely fast. When analyzing the video data, new insights were gained every time, we revisited the field through the sequences of recorded data [44]. Thus, the video observation technique is capable of capturing real time and continuous activities and hence of providing data, that allow us to study interactions between clinicians and technology in local time and place as well as over a period of time. Video observation provides the basis for insight, understanding and interpretation of the complexity of clinical work practice, as the complexity is recorded and analysis can be broken into smaller sequences and re-visited over and over again. These rich details cannot be achieved using other ethnographic observation methods. Besides, in contrast to other ethnographic methods – including personal observation - data from video observation allow us time and again to revisit the observation site and gain new insights, alone or together with the clinicians, with other stakeholders or with researchers, without having to physically return to the field.

In the same study as mentioned above [44], also the impact of the EHR on the exchange of information’s during ward-rounds was studied. At the wards, a number of work tasks went on simultaneously: communication between the patient and the clinician’s and between the clinician’s themselves, decisions on new medication and treatments, different clinical measurements etc. When subsequently analyzing the data, we recognized that our original focus on information flow was too narrow, because the EHR turned out to have a major impact on other aspects also, e.g. the organization of work. Thus, video observation has the advantage compared to personal observation and hand written notes that the recordings can be revisited time and again presenting revised research questions. Despite that the focus for the observation (by hand or video) is subjectively decided by the observer before or when the observation takes place, the recorded data are rich and embrace more than the initial focus.

When analyzing the data from an explorative study on medical secretaries work practice before the implementation of the EHR together with the involved secretaries, it became obvious that during interviews and personal observations conducted previously, only part of their work practice had been captured, partly because of the complexity, partly because of the routine and tacit nature of their work practice [16]. Thus, compared to data from personal observations, video data become a data repository allowing both contextual knowledge and the analysis process to be revisited and shared – and validated - with the involved clinical staff. As to validate the data generating process, the data repository permits the clinicians to access and discuss if the data recordings actually do represent their clinical work practice and further, if it represents the work practice that we, as researchers want to explore according to the study objective. Letting the clinicians themselves validate the video data, is a most reliable way of validating, e.g. the camera position in a clinical setting, because when a certain position is selected, others, which might also have an important meaning regarding the study objective, are left out.

Additionally, studying the video recordings in collaboration with the clinicians – and other relevant professionals in HIT development – provides an excellent basis for a dialog about understanding clinical work practice.

Overall, by providing us a thorough insight into the complexity of sociomaterial clinical work practice, the common benefits on video observation is insight into and a better understanding of the work practice of health care professionals.

### Lessons learned

Below we will present our most important lessons learned during three different phases in HIT- development:

the planning phase

the data collection phase

the data analysis and interpretation phase

We consider the planning phase the most important of all phases, because a well-planned study is crucial to the success of the following phases. Therefore, in this paper more attention is given to the planning phase compared to the other phases.

### The planning phase

The first and most important step in *any* study – no matter the methods and the techniques used - is the preparation of the study methodology, as this constitutes the “roadmap” from start to end [55]. However, a precondition for formulating the study methodology is clearly formulated objectives, as this decides the content of the methodology (e.g. the theoretical approach, the design, the data collection methods) [52,53,55-57]. Compared to other ethnographic data collection techniques, the video observation technique generates large amounts of data in a short period of time. It is therefore important to know exactly: why, where, whom, when and how to conduct the study, ie. to have a precisely formulated objective – and methodology. The fact that in “real life”, time, economic conditions, the study settings etc. very often are obstructive for an optimal methodology is not an excuse for the researchers not to focus on clear formulated objectives.

When conducting research – e.g. evaluation studies - in collaboration with external contracting authorities, the objective is often defined by the client beforehand. According to our experiences, such study objectives are often rather “loosely” formulated. An example is: “to provide an assessment of the clinical benefits of X system”. This objective might seem straightforward at first, but if not elaborated, a number of questions will inevitably arise later on in the study-process:

what is meant by “clinical benefits”?

from who’s point of view should the benefits be assessed? (clinician’s, management, patients - or other relevant actors in HIT development?)

by which indicators should clinical benefits be measured?

Thus, it is necessary to clarify the study objective to avoid any future misunderstandings and - what is also important - to adjust the study objective to meet “real life” constraints when it comes to economic conditions, time, study settings etc. However, a precondition for the researchers to be able to clarify a study objective is a thorough insight into the clinical context in which the study is going to take place. In a hospital setting, this will include:

number of wards

number of sections in each ward

number of employees – and professional groups to be involved in the study

the shifts (in a 24/7 setting) relevant to study

Some of this information can be achieved through homepages, literature and dialog/interviews with relevant key informants, but – based on our experiences - preliminary visits to the study locations to gain the needed insights through personal observation and interviews are mandatory. Based on the knowledge achieved through site visits etc., the researchers must then reach an agreement on clear and measurable objectives to prevent major problems and misunderstandings later on in the process. First then, the additional elements of the methodology (e.g. design and data collecting methods) should be formulated.

When the objective indicates that the video observation technique is beneficial, it is important to reflect and decide on the kind of video observation (Figure 1). However, this can often be read from the (clearly and precisely) formulated objectives, as theformulation of these often (directly or indirectly) tells, if the researcher should interact with clinical practice or not.

The next step in the planning phase is to decide on:

1. the overall perspective: specific work practices, certain professional groups or…?

2. the focus: e.g. *which* specific clinical work practice performed by *which* professionals at *which* wards at *which* time by *which* researchers?

3. which camera angels should be used: e.g. should the video be fixed or roving - and does the setting (e.g. space, patients, work-procedures) allow a camera to be placed in the optimal position?

4. what is left out by the decisions taken in 1, 2 and 3 - and how does this impact the results?

5. how many researchers will do the recordings?

6. for how long time (hours, days, weeks) to record?

The list above is not exhaustive, but it comprises the issues found most important during our studies. Common to all questions are that the answers are closely linked to the study objectives. Thus, when answering the questions, the study objective must simultaneously be readdressed.

An important step during the planning phase – which should be taken as soon as the study objective is formulated - is getting permission to do video recordings within the clinical setting, ie. at the wards. Our experience is that a well prepared study and clear objectives are imperative for achieving the trust and confidentiality of the management necessary to gain permission to do video recordings within a ward.

However, having gained permission from the management does not imply that we walk straight into e.g. a hospital ward and start recording. To establish trust and willingness to participate in the study among the clinical staff is an important part of the planning phase. Thus, at preliminary visits at we inform the clinicians on the study objective, what our presence mean to their daily work, what is expected of them, etc. This is an issue, which is also stressed by Heath et al. [47]. During our video observation studies, we have only met few clinicians who have declined to be video taped, and we have met none, who - after having been well informed about the study objectives and methods – have insisted on not participating. However, prior to data collection, informed consent should be obtained from all staff members who are to be observed cf. the section on ethical considerations.

When the planning phase is completed, the next steps in the study process should be far less time consuming – given that the study methodology is well prepared and all the precautions mentioned in this section have been taken.

### The data collection phase

As mentioned in the section on ethical considerations, a very important consideration, when video-recording within a clinical setting, is how to avoid video recording the patient – and especially the patient’s face. When recording within a ward, the optimum solution to this is to place the camera at the headboard of the bed. From this angle, it is possible to capture most activities going on in the room without recording the patient’s face. It can be difficult to avoid recordings of patients, when e.g. following a clinician up and down the corridors with a roving camera while he/she is performing a ward-round. If a patient’s face by accident is video-recorded, we delete that sequence.

During recordings, it is important fully to adhere to the methodology formulated in the planning phase. This means that when the observation method decided on is e.g. non-participant observation (fly at the wall), the researcher should refrain from interfering with the clinicians and the clinical work practice. If not, the data-validity is compromised, as the data gathered will be influenced by the researcher’s interaction. If the video observation method is participant observation, the researcher may ask the clinicians to reflect on their own practice (fly in the eye). This may provide a clarification and elaboration of socio-material activities, issues and situations while video recording, and thereby provide more in depth and elaborated information of e.g. reasons behind specific clinical work practice. Information, that otherwise would remain tacit knowledge and/or stickyinformation [58].

**Figure 2:**
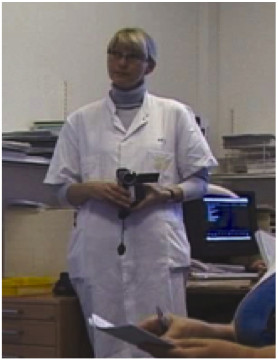
**Position of the researcher and the camera.** Figure 2 shows one the authors (Anna Marie Høstgaard) dressed as the clinicians and the size and position of the camera. The example is from the evaluation of the GEPKA project [45].

It is our experience that when clinical staff is video recorded, they often try to perform certain tasks according to “what the books tell”, instead of how they themselves have appropriated work practice to fit the clinical context and the resources available. Thus, our presence *does* affect the way they behave when performing their daily clinical tasks. However, the more and the better the clinicians have been informed on the study objective beforehand, the better we have been able to establish contact and trust - which is a precondition for diminishing this bias. Thus, after a while (often only one-two hours), they seem to be less affected by our presence. This view is supported by Nøhr et al. [19]. We have found that it is important to dress up like the people we observe, e.g. in white coats and to use small video-recorders and to hold them in the least possible eye-catching position in order to attract as little attention as possibly (Figure 2). 

### The data analysis and interpretation phase

Analyzing video-recorded observation data differs from analyzing data collected through other ethnographic methods by the huge quantity of data – both visual and audial. If the study has *not* been conducted from a fixed, clear and precise methodology, problems often arise during this phase, because of the researchers loosing focus and missing the “red thread” when navigating through the (high time-consuming) analysis phase. Part of - or in worse case - all the collected data can then prove to be useless, because they prove irrelevant to the study objective. During one of our studies, the objective was revised by external contracting authorities half way through the study. Originally, the objective was to assess the clinical benefits of the fully implemented system from the clinician’s perspective. This was changed into an assessment of the clinical benefits of the implementation of only the first part of the system. This meant that a substantial part of our baseline data became irrelevant [44].

**Figure 3:**
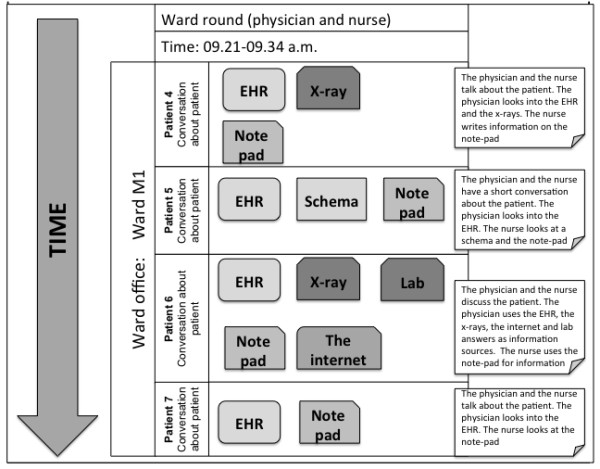
**Chart providing information on activities in a ward office.** Figure 3 shows a chart providing information on activities in a ward office, when a physician and a nurse are preparing for the ward round: at what time (time-line to the left): which artefacts are used: who are using them: where it takes place and patients involved [37].

If, on the contrary, the data have been collected from a well-planned methodology, the researchers still have a large amount of data – but they also have an overall plan for the analysis phase. We have developed a systematic method including three major steps on how to manage this phase:

Step 1: Create an overview of all the data (often many hours of recordings) in order to identify the sequences relevant according to the study objectives. Look through all the video recordings and at the same time outline the work practice activities in a chart: what is going on: when: which technologies are used: who is using them: and where does the activity take place. Figure 3 shows an example of a work practice analysis of a physician’s ward-round.

The chart allows us to identify which data sequences are the most relevant according to the study objective, as a large quantity of the collected data always prove not to be important (e.g. walking down the corridors, waiting for the results of tests, the clinicians having breaks).

Step 2: The data-sequences identified as relevant according to the study objective are then analyzed in depth. This includes transcribing and mapping the clinicians – as well as other relevant actors – actions. We will not go into details with this process, as other researchers have already done this [10,47].

Step 3: The last major step is to interpret the results from step 2. According to our experiences, clinical work practices are very hard to compare across contexts, settings and time (e.g. before and after implementation of HIT), because of the individual nature of clinicians’ work. Besides, also the patients involved are different. Therefore comparing clinical work practice should be done with great caution. We share this view with other researchers [44,59-62].

When interpreting video-data – as any qualitative data – it is very important for the researcher to be explicit and transparent about the study paradigm as this shows the researchers ontological position. As mentioned in the method section, the same data can be interpreted differently depending of the ontological position held by the researcher.

## Discussion

### Studies on successful HIT development and implementation

During the history of HIT, numerous studies have focused on identifying barriers and/or factors for success in HIT development and implementation Despite an awareness on the fact that the complexity and unpredictability in clinical work practice involves a great challenge with regard to set up common success and/or failure factors, more studies claim that common themes across contries and HIT systems can be identified [63-66]. However, according to Berg, even when there is a total agreement on the objective of an HIT implementation, “there exist no simple formula for success” [7] p.146, because of the complexity of the sociomaterial work practices. We fully agree with Berg on his view on setting up definite success criteria for HIT success. However, we argue – with Bergs considerations in mind – that a precondition for the development of more successful HIT systems in the future is more research into what contributes to successful HIT development and implementation - ie. more research aiming at a better understanding of the complexity in clinical work practice. According to our experiences, an important tool in achieving this goal is the video observation technique.

The views presented in this paper are based on more than ten years of scientific studies using video observation for data collection *within* different health care settings – ie. at hospital wards. While employing the technique, we have simultaneously improved it. This process has provided us a thorough insight into when and where video observation is beneficial to HIT development – and when it is not. We find this a solid foundation for passing on our experiences.

### Strengths and weaknesses

So far, other researchers have only studied clinical work practice by traditional ethnographic methods or in usability laboratories or clinical set-ups. When studied in the latter, e.g. unforeseen disruptions and communication challenges are hard to imitate no matter how realistic the artificial clinical setting is set up [36-39]. When video observation has been used within healthcare settings, it has been for studies of specific clinical procedures [40,41] for studies of social processes between different clinical professionals [42], or for studying the impact of video on the clinicians [43]. The experiences presented on clinical work practice in this paper are all based on studies using the video observation technique *within* healthcare settings –ie. at hospital wards. This permits us to explore *context dependent* clinical sociomaterial work practices in real time and place.

As for all other types of qualitative data collection methods and techniques involving the researcher, video observation will not lead to the “truth” about how clinical work is practiced. However, data collection using video observation has many advantages in providing an understanding of clinical work. Roughly speaking, video observation allows you a) to be a fly on the wall – record what take place - and then later discuss the activities with the clinicians or b) to follow the clinicians and encourage them to elaborate on their actions while in action – this is especially relevant if there will be no chance to discuss the video recording with them later on.

It is important for us to stress that our concern - when studying clinical sociomaterial work practice from a hermeneutical point of view - is to broaden our insight and understanding of local situated work practices. In several of the cases included in this paper, we have video recorded work practices one day before- and one day after the implementation of a new health information technology (Table 1). This provides insight into concrete sociomaterial work practices and hence into whether the associated interactions between the clinicians and the technology are in balance or dysfunctional. We are well aware of the fact that this does not provide an exact foundation for comparisons or for generalizations across settings or even within settings in the positivistic sense - e.g. to make claims of statistical significance from our results.

We are also aware of the fact that formulating a clear and measurable objective is not only important in video observation studies, as this is crucial to *any* scientific study. However, the reason why we have stressed this issue repeatedly in this paper is the fact that the greatest problems that we have experienced in our video observation studies have all been due to imprecise study objectives – or objectives being changed during the study period. In one particular case, this meant that part of our baseline data became irrelevant [44]. Thus, imprecise objectives might cause misunderstandings between different actors later on in the HIT development process or problems of a methodological nature in the analysis phase. Compared to other ethnographic methods and techniques, video observation generates large amounts of data in a short period of time – both visual and audial. Therefore, to avoid collecting irrelevant data, it is important to have a clear strategy for the study, ie. a clearly formulated methodology. Also, gaining access to video recording within hospital settings rest primarily on the study-objective being clearly formulated and on the overall methodology being transparent, as this is the basis for convincing hospital management and IT-board that the study is worthwhile pursuing.

The experiences presented in this paper are based on a hermeneutical point of view, which means that the data collection has primarily been performed with a view to understand and interpret clinical work practice. However, the video observation method has also proven beneficial within the positivistic paradigm as shown in on of our cases (Table 1, case nr. 1). In this case, a before and after *time* study was performed using the time-function provided with the video technique. Bearing all the usual methodological considerations in mind, we argue, that the video observation technique has great potentials also within other study-paradigms - including the positivistic.

During recent years the focus in HIT development has moved from mainly concentrating on hospital technologies towards patient centered healthcare solutions, e.g. outpatient clinics, telemedicine, primary care for chronically and/or elderly patients in their own homes [67,68] We argue that the video observation technique will also provide benefits within these areas.

## Conclusions

By employing and further developing the video observation technique, we have found, that it is superior to other ethnographic research techniques when it comes to disclosing the complexity in clinical sociomaterial work practice. We have also found that by using the video observation technique *within* the healthcare context (ie. at hospital-wards) the insights gained of the clinical sociomaterial work practices are realistic. Additionally, that the video data generated this way provide a solid basis for dialog between clinicians and IT-professionals and other professional groups involved in HIT development. Overall, video observation can teach us to know and to better understand the users work practice, and therefore, to know better the user requirements and needs for HIT. From a socio-technical perspective this is a precondition for the development of more successful HIT systems in the future.

The most important lessons learned during our studies are, firstly, that a well considered methodology and clear formulated objectives are extremely important to stay focused and not miss the “red thread” when analyzing the large amount of data generated when using the video observation technique. Secondly, that the video observation technique primarily should be used for studies of specific clear identified clinical work practice within delimited clinical settings, avoiding the temptation to study a wide range of clinical work practice at different clinical settings as this results a great challenge time wise.

It is our hope that this paper will inspire other researchers to embark on using video observation technique and to engage in a debate on strengths and limitations of the various data collection methods in HIT studies.

## Endnotes

^a^DaCHI was until 2012 entitled: V-CHI (Virtual Centre of Health Informatics).

^b^Clinical set-ups: Laboratories equipped with hospital beds and all other clinical artefacts belonging to a hospital ward.

## Competing interests

Both authors declare that they have no competing interests.

## Authors' contributions

Both authors contributed to the conceptual design of this study. AMH was responsible for writing the manuscript. PB was involved in drafting the manuscript and revising it critically for important intellectual content. Both authors have read and approved the final manuscript.

## Pre-publication history

The pre-publication history for this paper can be accessed here:

http://www.biomedcentral.com/1472-6947/12/91/prepub
